# Methotrexate-induced nausea in the treatment of juvenile idiopathic arthritis

**DOI:** 10.1186/s12969-017-0180-2

**Published:** 2017-06-19

**Authors:** Sonja Falvey, Lauren Shipman, Norman Ilowite, Timothy Beukelman

**Affiliations:** 1 University of Mississippi School of Pharmacy, Oxford, USA; 2 Division of Pediatric Rheumatology, University of Alabama Birmingham, Birmingham, USA; 3 Children’s Hospital at Montefiore, Albert Einstein College of Medicine, Bronx, USA

**Keywords:** Methotrexate, MTX, Nausea, Vomiting, Juvenile idiopathic arthritis, JIA

## Abstract

**Background:**

Methotrexate is the most commonly used disease modifying antirheumatic drug in the treatment of juvenile idiopathic arthritis and can be effective in controlling disease in many patients.

**Main body:**

A significant proportion of patients experience nausea and vomiting induced by methotrexate therapy, which can lead to decreased quality of life and discontinuation of treatment with methotrexate. Many strategies have been employed in attempts to reduce methotrexate-induced nausea, including folate supplementation, switching from oral to subcutaneous methotrexate, anti-emetic therapy, behavioral therapy, and others. Anticipatory nausea can be difficult to treat, making primary prevention of nausea with anti-emetics an attractive approach.

**Conclusion:**

Understanding the prevalence and impact of methotrexate-induced nausea, as well as potentially effective interventions, may help maximize the therapeutic benefits of methotrexate.

## Background

Juvenile idiopathic arthritis (JIA) is defined by the International League of Associations for Rheumatology as arthritis of an unknown etiology that persists for at least 6 weeks in children under the age of sixteen [1] and is the most common rheumatic disease in childhood [2]. Effective and timely treatment is necessary to ensure present and future quality of life for children with JIA [2].

After initially serving in the 1950’s as a treatment for acute leukemia and other malignancies, methotrexate (MTX) was shown to be efficacious in the treatment of children with chronic arthritis. This was first published in 1992 in the breakthrough US-USSR controlled study [3], with subsequent controlled trials and observational studies supporting its findings [4–6]. MTX became the most commonly used disease modifying antirheumatic drug (DMARD) to treat JIA and remains the most common to the present day [7–9]. Treatment with MTX has the infrequent but serious potential to cause significant hepatotoxicity or bone marrow suppression; however, the frequency and consequences of MTX-induced nausea likely have a greater clinical impact in practice [10, 11].

The gastrointestinal (GI) adverse effects of MTX, most commonly MTX-induced nausea, frequently lead to non-adherence or discontinuation of this otherwise effective and low-cost drug. Different strategies have been employed to diminish the adverse GI effects of MTX, and these have met with variable success. In this paper, we aim to review the published data about MTX-induced nausea in the treatment of JIA, as well as possible management approaches including the use of newer anti-nausea medications.

## Frequency and significance of MTX-induced nausea

Although nausea is one of the most frequent adverse effects of MTX, its prevalence and epidemiology in children with JIA has not been completely characterized. One cross-sectional descriptive study performed in the Netherlands reported the prevalence of MTX intolerance owing to GI effects as determined by the Methotrexate Intolerance Severity Score (MISS) to be 50.5% in a cohort of 297 children with JIA. In the same study, 191 (64%) patients experienced MTX-induced nausea, and 81 (27%) reported MTX-associated vomiting [11]. A cohort study of new MTX initiators in the Netherlands reported a 1 year incidence of 42% for MTX intolerance owing to GI effects [12]. An international randomized clinical trial of two doses of methotrexate involving 80 patients with polyarticular JIA reported nausea in 21% and vomiting in 11% of patients [6]. In a survey of 49 adolescents in England with inflammatory arthritis who were receiving MTX, 73% reported nausea and 43% reported vomiting [10]. In a multicenter, double blind, placebo-controlled crossover clinical trial that analyzed the use of low-dose oral methotrexate in 88 children with JIA, nausea was reported in 28% [4]. One survey of 171 mothers of children with JIA reported that almost a third of the JIA patients felt sick every week and 15% vomited every week after taking MTX [13]. A recent survey regarding MTX related nausea and vomiting in patients with JIA was completed by 84 pediatric rheumatology care providers in the United Kingdom (UK). Approximately one-half of the respondents reported the development of nausea and vomiting associated with MTX in greater than 25% of their patients during the first year of treatment with MTX [14].

In summary, there is a wide range of reported rates of MTX-induced nausea in the treatment of JIA (Table 1). The minimum estimate appears that at least 1 out of 5 patients, and perhaps as many as 3 out of 4 patients, suffer from MTX-induced nausea, depending on the definition of nausea used and the method of ascertainment. Importantly, MTX-induced nausea has been associated with significantly decreased quality of life in children with JIA [13] and the presence of significant GI symptoms in general has been associated with worse quality of life in children with JIA [15].

**Table 1:** Published prevalence rates of GI adverse effects associated with MTX in the treatment of JIA

Author	Year published	Study design	Number of subjects	Method of ascertainment	MTX-intolerance due to GI effects	Nausea with MTX	Vomiting with MTX	Anticipatory nausea
Bulatovic	2011	Cross-sectional	297 patients	MISS	51%	64%	27%	9%
van Dijkhuizen	2015	Cohort study of new MTX initiators	142 patients	MISS	42% (1 year incidence)	(n/a)	(n/a)	(n/a)
Ruperto	2004	Randomized clinical trial of MTX	80 patients	Direct questioning	(n/a)	21% (monthly)	11% (monthly)	(n/a)
Patil	2014	Survey	49 patients	Questionnaire	(n/a)	73%	43%	41%
Woo	2000	Randomized clinical trial of MTX	88 patients	Direct questioning	(n/a)	28% (monthly)	(n/a)	(n/a)
Mulligan	2013	Survey	171 parents	Questionnaire	(n/a)	~32% (weekly)	15% (weekly)	25% (weekly) >35% (any)
Amin	2015	Survey	84 physicians	Questionnaire	(n/a)	~25% in first year		(n/a)

*MTX* methotrexate, *GI* gastrointestinal, *MISS* methotrexate intolerance severity score

MTX-induced nausea often results in discontinuation of MTX and consequent initiation of other medications. In the open label screening phase of a randomized study of MTX dosing, 9 of the 25 (36%) children who discontinued low-dose MTX did so owing to GI complaints [6]. In a retrospective review of 58 children with JIA who received leflunomide, 39 (67%) of them had discontinued MTX because of nausea [16]. In the recent survey of pediatric rheumatology care providers in the UK, it was estimated that greater than 10% of patients who initiated treatment with biologic agents did so because of intolerance of MTX-induced nausea [14].

## Clinical factors associated with MTX-induced nausea

A few clinical factors have been reported to be associated with an increased incidence of MTX-induced nausea. A survey of patients with inflammatory arthritis demonstrated an inverse relationship between age and MTX-induced nausea and vomiting; adolescent patients had over six times higher odds of reporting nausea when compared to adult patients (odds ratio (OR) 6.31 [95% confidence interval (CI) 2.38-16.75]). This study also found that patients who were taking MTX for more than 1 year had almost four times higher odds of developing nausea compared to patients on MTX for only 3–11 months (OR 3.86 [95% CI 1.71-11.79]) [10].

## Anticipatory nausea associated with MTX

Anticipatory nausea is a known psychological adverse effect of MTX that develops through classical conditioning. Nausea may be triggered by cues such as seeing MTX or simply hearing the word spoken [17]. Although anticipatory nausea is well-studied in cancer patients receiving chemotherapy treatment and has a reported frequency of 30% [11], it has only been assessed in a few studies of children with JIA (Table 1). In the aforementioned survey of adolescents who receive MTX, anticipatory nausea was reported in 41% of the participants [10]. In the aforementioned survey of mothers of children with JIA receiving MTX, nearly 25% reported anticipatory nausea with every weekly dose of MTX and more than 35% reported any anticipatory nausea [13]. The previously mentioned cross-sectional study performed in the Netherlands reported 9% of patients experienced anticipatory nausea. Interestingly, among patients who were considered to be MTX intolerant according to the MISS, 18.7% reported anticipatory nausea, whereas no MTX tolerant patients reported anticipatory nausea. Anticipatory nausea can be difficult to treat and may result in non-adherence and treatment discontinuation. The best strategy appears to be to avoid the development of anticipatory nausea in the first place [18].

## Assessing MTX-induced nausea

Bulatovic et al. developed the Methotrexate Intolerance Severity Score (MISS) instrument to measure MTX intolerance owing to GI-related symptoms [11]. The MISS includes items about stomachache, nausea, vomiting, and behavioral complaints and assesses both direct MTX effects as well as anticipatory or associative effects. With physician’s opinion serving as the gold standard, the validation study determined that 90% of patients were correctly classified as being MTX intolerant using the MISS [11]. The MISS has subsequently been used to study adults with rheumatoid arthritis and psoriatic arthritis [19].

Another instrument to evaluate GI-related symptoms in JIA is the Gastrointestinal Symptom Scale for Kids (GISSK) [15]. This instrument was developed to assess for GI-related symptoms in general, not symptoms specifically related to treatment. It assesses dyspepsia, upper and lower abdominal pain, nausea, diarrhea, constipation, vomiting, and anorexia. The validation study identified the presence of GI symptoms in the majority (58%) of children with JIA, with 22% having nausea [15]. The GISSK does not specifically assess the temporal relationship between symptoms and medication administration, including anticipatory symptoms. Given its targeted development, the MISS is likely a superior method to assess MTX-associated nausea and intolerance in children with JIA, although the two instruments have not been compared directly in a study.

### Folate supplementation for MTX-induced nausea

Folic acid (vitamin B9) and folinic acid (5-formyl tetrahydrofolate) are forms of folate that can be taken orally to attempt to reduce MTX toxicities. Both are able to function in biosynthetic pathways that are independent of the dihydrofolate reductase enzyme that MTX inhibits as part of its mechanism of action. It is thought that folic acid and folinic acid help to reduce the adverse effects of MTX by replenishing depleted intracellular folate levels that occur in hepatocytes and peripheral blood lymphocytes of patients treated with MTX. However, because the exact mechanisms of action of MTX in the treatment of arthritis are unknown, there is some concern that folate supplementation may reduce the efficacy of MTX, especially if the antirheumatic effects are mediated partially through folate antagonism [20].

Folate supplementation in JIA has been examined in a few published studies. In a randomized double blind placebo controlled crossover trial of 19 children with juvenile arthritis receiving MTX, each subject received either folic acid or placebo for 12 weeks to observe the impact of folic acid supplementation on the efficacy of MTX. It was determined that 1 mg/day of folic acid did not result in increased disease activity. The effects of folic acid on MTX-induced nausea were not assessed. A retrospective chart review of 43 children with juvenile arthritis who initiated folinic acid supplementation following the development of adverse effects of MTX demonstrated that folinic acid significantly reduced GI-associated symptoms from a mean of 1.09 episodes per patient-year to a mean 0.29 episodes per patient-year [21]. When starting MTX for the first time, 67% of respondents in the UK pediatric rheumatology provider survey reported always or often (>50% of the time) concurrently starting folic acid [14].

Folate supplementation in adult patients receiving methotrexate for rheumatoid arthritis was the subject of a recently published Cochrane Review [20]. A meta-analysis of 6 randomized controlled trials revealed a significant risk reduction for nausea, vomiting, or abdominal pain (RR 0.74 [95% CI 0.59 to 0.92]) with the use of folate supplementation compared to placebo. Folate supplementation did not appear to have any effect on the efficacy of MTX as measured by tender and swollen joint counts and physician global assessment scores. The results of the review did not show any definite clinical advantage of one form of folate supplementation over the other. However, because folic acid is considerably less expensive than folinic acid, folic acid is probably the more cost-effective therapy [20].

### Subcutaneous MTX for nausea reduction

A common perception among rheumatologists is that the oral route of MTX administration is more likely to result in nausea and vomiting compared to the parenteral route, but the published evidence is challenging to interpret. In support of the common perception, a published retrospective review of 61 children with JIA treated with oral MTX identified 11 patients who were subsequently switched to SQ MTX because of nausea. Of these 11 patients, 9 (82%) experienced complete resolution of nausea and the other 2 experienced less severe nausea and were able to continue MTX therapy [22].

On the other hand, some studies have found the prevalence of MTX-induced nausea and vomiting to be greater in patients receiving parenteral MTX compared to those receiving oral MTX. The aforementioned survey of adolescents and adults with inflammatory arthritis reported that 77% of patients on parenteral MTX experienced nausea compared to 37% in the oral group (*p* < 0.0001) [10]. In a survey of 171 mothers of children with JIA, children receiving SQ MTX had higher adjusted odds of feeling sick before (OR 2.33 [95% CI 0.96-5.66]) but not necessarily after (OR 1.50 [95% CI 0.69 to 3.26]) MTX administration compared to those receiving oral MTX. Children receiving SQ MTX also had higher adjusted odds of vomiting after administration (OR 3.75 [95% CI 1.28-11.06]) compared to oral MTX [13]. Bulatović et al. found that the prevalence of nausea in patients receiving parenteral MTX was 61 versus 41% for oral MTX (*p* = 0.002), and patients receiving parenteral MTX had a higher adjusted odds of being MTX intolerant according to the MISS questionnaire compared to those receiving oral MTX (OR 1.9 [95% CI 1.01-3.58]) [11]. Importantly, these studies shared a major limitation: one of the primary reasons for children to switch from the oral to SQ route of administration is initial GI intolerance of the oral route, and this bias was not addressed.

A recently published cross-sectional study was conducted at several centers in Germany with the goal of assessing how the route of MTX administration affects MTX intolerance in patients with JIA [23]. The MISS questionnaire was used to compare MTX intolerance in 2 patient groups: 95 patients receiving oral MTX with no history of SQ MTX use and 46 patients receiving SQ MTX with no history of oral MTX use. The median methotrexate dose for the exclusively PO group and the exclusively SQ group was comparable at 11.8 mg/m2/week and 11.6 mg/m2/week, respectively. A greater proportion of patients receiving SQ MTX had MTX intolerance according to the MISS instrument compared to those receiving oral MTX (43 vs. 29%, OR: 3.4[95% CI 1.2-10.0]); however, there was no significant difference in the prevalence of nausea or vomiting between the 2 groups, and the significant differences between the groups lie in the behavioral components of intolerance.

## Anti-emetics for MTX-induced nausea

Ondansetron is a 5-HT_3_-receptor antagonist that is often used to treat nausea induced by chemotherapy in cancer patients. Ondansetron is an effective first-line antiemetic for children treated with chemotherapy and has been shown to be significantly more effective than metoclopramide and chlorpromazine in comparative studies [24]. More recently, ondansetron has been increasingly used to treat other causes of nausea. For example, a Cochrane Review analyzed the results of studies of ondansetron in the emergency department for vomiting related to acute gastroenteritis in children and found significant reductions in hospitalization rates and in the need for intravenous fluids [25]. In addition, ondansetron has been shown to be very well tolerated by children in the treatment of nausea for various indications. [18, 24–26].

Perhaps more relevant to the issue of MTX-induced nausea in children with JIA, ondansetron appears effective in pediatric patients with Crohn disease and MTX-induced nausea. In a retrospective study, researchers evaluated the occurrence of MTX-induced nausea among children with Crohn disease who received ondansetron pre-medication with the first dose of SQ MTX compared to those who did not initially receive ondansetron pre-medication [27]. Among the 50 children who initially received ondansetron pre-medication, only 1 (2%) experienced nausea in the 3 months after starting MTX, compared to 6 (60%) among the 10 children who did not initially receive ondansetron premedication (*p* < 0.001). Four of the 6 children who developed nausea without ondansetron premedication subsequently received ondansetron prior to weekly MTX doses and had no more complaints. The authors concluded that ondansetron was effective therapy for MTX-induced nausea associated with parenteral treatment in children with Crohn disease [27].

Ondansetron has also been shown to be effective in treating MTX-induced nausea in adults with rheumatoid arthritis. In an observational study, 9 adults with rheumatoid arthritis were given ondansetron because they had severe nausea that persisted despite receipt of the anti-emetic metoclopramide and switching MTX from oral to intramuscular administration [28]. Following initiation of ondansetron, the intensity and duration of nausea declined dramatically in all patients, and this improvement continued throughout the 24 weeks of follow-up.

Some pediatric rheumatologists are using ondansetron in clinical practice. Among the 84 respondents to the UK provider survey, 21% always or often (>50% of the time) start an anti-emetic concurrently when starting MTX for the first time. When prescribing an anti-emetic, ondansetron was the first choice for 88% of respondents [14].

Dosing recommendations for the use of ondansetron for the prevention of nausea and vomiting associated with emetogenic medications are provided by the manufacturer. For children ages 4 to 11 years, the recommended dose is 4 mg orally given 30 min prior to emetogenic medication. For children age 12 years or older, the recommended dose is 8 mg orally given 30 min prior to emetogenic medication [29]. Patients may take 1–2 additional doses of ondansetron every 8 h as needed for post-MTX nausea and vomiting.

### Behavioral interventions for MTX-induced anticipatory nausea

Behavioral interventions may help increase tolerance to MTX and reduce the occurrence of associative and anticipatory nausea. In a retrospective chart review of children with JIA, investigators reported outcomes for 10 patients who were referred to a pediatric psychologist for behavioral therapy for anticipatory nausea (*N* = 6) and anxiety (*N* = 9) related to MTX [17]. Behavioral therapy was adapted to age. Children less than 10 years old received the “Magic Box” method based on systemic desensitization by distraction, and older children received cognitive behavioral therapy. The behavioral therapy intervention was found to be fully effective in 5 children (50%), moderately effective in 2 children (20%), and not effective in 3 children (30%) [17].

## Alternative therapies to MTX

MTX-induced nausea can be intolerable and may lead to medication discontinuation. Fortunately for patients, there are alternative therapies available in cases of MTX intolerance.

Leflunomide is a non-biologic agent that is effective for the treatment of JIA. Leflunomide was directly compared to MTX in a randomized, blinded, placebo-controlled clinical trial of 94 children with polyarticular JIA who were naïve to both drugs [30]. At 16 weeks following randomization, a greater proportion of children in the MTX group achieved an ACR Pedi 30 response compared to the leflunomide group (89 versus 68%; *p* = 0.02). This observed result may have been partially attributable to differences in medication dosing, and other measures of effectiveness were similar between the two medications. In observational studies, children who initiated treatment with leflunomide owing to intolerance of MTX generally demonstrated good clinical responses and were not likely to discontinue leflunomide due to nausea [16, 30]. Taken together, these findings suggest that MTX may be more effective, but leflunomide is a possible alternative in the event that a patient cannot receive MTX for various reasons [30]. Because it is more expensive than MTX [31] and less well-studied, in clinical practice leflunomide is generally reserved for patients who have failed MTX owing to either intolerance or inefficacy [7].

Newer biologic agents, in particular the tumor necrosis factor inhibitors (TNFi), have been shown to be highly effective in the treatment of JIA [32]. MTX intolerance has been addressed in many patients by stopping MTX and initiating treatment with TNFi, although this practice has not been well-studied. A majority of the respondents (63%) in the UK survey reported more than 10% of their patients were switched from MTX to a biologic in response to MTX associated nausea and vomiting [14].

In contrast to current recommendations to use MTX prior to biologics [32], it appears that in clinical practice many patients receive TNFi without a trial of MTX use according to a recently published study of pharmacy records from the United States [8]. The reasons for this practice were unclear from this study, but the anticipated relatively high rate of MTX intolerance may play a role. Because TNFi and other newer therapeutic agents are considerably more expensive than MTX, increased tolerance of MTX among patients in whom it is effective could represent a significant cost-savings. In addition, because the combination of MTX and a biologic agent is generally more effective than a biologic agent alone [32], decreasing the impact of MTX-associated nausea would likely also benefit patients whose arthritis is refractory to MTX monotherapy.

## Conclusions

MTX is an effective, relatively safe, and low-cost treatment for children with JIA, but its use is often limited by significant nausea. Various management approaches have been attempted and there is no clear single preferred method. Folate supplementation should likely be taken by all patients receiving MTX to help prevent nausea, as well as potentially counteract other adverse effects of MTX. Co-medication with anti-emetics, such as ondansetron, appears to be a highly effective approach, and behavioral therapies may be helpful with anticipatory nausea. There are alternative therapies to MTX in cases of intolerance, but initial prevention of intractable nausea is likely the best approach.

## Abbreviations

DMARD: Disease modifying antirheumatic drug
GI: Gastrointestinal
GISSK: GI symptom scale for kids
JIA: Juvenile idiopathic arthritis
MISS: Methotrexate intolerance severity score
MTX: Methotrexate
PO: By mouth
SQ: Subcutaneous
TNFi: Tumor necrosis factor inhibitor
UK: United Kingdom

## References

[1] Petty RE, Southwood TR, Manners P, Baum J, Glass DN, Goldenberg J (2004). International league of associations for rheumatology classification of juvenile idiopathic arthritis: second revision, Edmonton, 2001. J Rheumatol.

[2] Gowdie PJ, Tse SM (2012). Juvenile idiopathic arthritis. Pediatr Clin N Am.

[3] Giannini EH, Brewer EJ, Kuzmina N, Shaikov A, Maximov A, Vorontsov I (1992). Methotrexate in resistant juvenile rheumatoid arthritis. Results of the U.S.A.-U.S.S.R. double-blind, placebo-controlled trial. The Pediatric rheumatology collaborative study group and the cooperative Children's study group. N Engl J Med.

[4] Woo P, Southwood TR, Prieur AM, Dore CJ, Grainger J, David J (2000). Randomized, placebo-controlled, crossover trial of low-dose oral methotrexate in children with extended oligoarticular or systemic arthritis. Arthritis Rheum.

[5] Klein A, Kaul I, Foeldvari I, Ganser G, Urban A, Horneff G (2012). Efficacy and safety of oral and parenteral methotrexate therapy in children with juvenile idiopathic arthritis: an observational study with patients from the German Methotrexate registry. Arthritis Care Res (Hoboken).

[6] Ruperto N, Murray KJ, Gerloni V, Wulffraat N, de Oliveira SK, Falcini F (2004). A randomized trial of parenteral methotrexate comparing an intermediate dose with a higher dose in children with juvenile idiopathic arthritis who failed to respond to standard doses of methotrexate. Arthritis Rheum.

[7] Beukelman T, Ringold S, Davis TE, DeWitt EM, Pelajo CF, Weiss PF (2012). Disease-modifying antirheumatic drug use in the treatment of juvenile idiopathic arthritis: a cross-sectional analysis of the CARRA registry. J Rheumatol.

[8] Mannion ML, Xie F, Curtis JR, Beukelman T (2014). Recent trends in medication usage for the treatment of juvenile idiopathic arthritis and the influence of tumor necrosis factor inhibitors. J Rheumatol.

[9] Hashkes PJ, Becker ML, Cabral DA, Laxer RM, Paller AS, Rabinovich CE (2014). Methotrexate: new uses for an old drug. J Pediatr.

[10] Patil P, Parker RA, Rawcliffe C, Olaleye A, Moore S, Daly N (2014). Methotrexate-induced nausea and vomiting in adolescent and young adult patients. Clin Rheumatol.

[11] Bulatovic M, Heijstek MW, Verkaaik M, van Dijkhuizen EH, Armbrust W, Hoppenreijs EP (2011). High prevalence of methotrexate intolerance in juvenile idiopathic arthritis: development and validation of a methotrexate intolerance severity score. Arthritis Rheum.

[12] van Dijkhuizen EH, Bulatovic Calasan M, Pluijm SM, de Rotte MC, Vastert SJ, Kamphuis S (2015). Prediction of methotrexate intolerance in juvenile idiopathic arthritis: a prospective, observational cohort study. Pediatr Rheumatol Online J.

[13] Mulligan K, Kassoumeri L, Etheridge A, Moncrieffe H, Wedderburn LR, Newman S (2013). Mothers' reports of the difficulties that their children experience in taking methotrexate for juvenile idiopathic arthritis and how these impact on quality of life. Pediatr Rheumatol Online J.

[14] Amin TS, Shenton S, Mulligan K, Wedderburn LR, Wood M, VanRooyen V (2015). Strategies for the prevention and management of methotrexate-related nausea and vomiting in juvenile idiopathic arthritis: results of a UK paediatric rheumatology prescriber survey. Rheumatology (Oxford).

[15] Brunner HI, Johnson AL, Barron AC, Passo MH, Griffin TA, Graham TB (2005). Gastrointestinal symptoms and their association with health-related quality of life of children with juvenile rheumatoid arthritis: validation of a gastrointestinal symptom questionnaire. J Clin Rheumatol.

[16] Foeldvari I, Wierk A (2010). Effectiveness of leflunomide in patients with juvenile idiopathic arthritis in clinical practice. J Rheumatol.

[17] van der Meer A, Wulffraat NM, Prakken BJ, Gijsbers B, Rademaker CM, Sinnema G (2007). Psychological side effects of MTX treatment in juvenile idiopathic arthritis: a pilot study. Clin Exp Rheumatol.

[18] Zoubek A, Kronberger M, Puschmann A, Gadner H (1993). Ondansetron in the control of chemotherapy-induced and radiotherapy-induced emesis in children with malignancies. Anti-Cancer Drugs.

[19] Calasan MB, van den Bosch OF, Creemers MC, Custers M, Heurkens AH, van Woerkom JM (2013). Prevalence of methotrexate intolerance in rheumatoid arthritis and psoriatic arthritis. Arthritis Res Ther.

[20] Shea B, Swinden MV, Tanjong Ghogomu E, Ortiz Z, Katchamart W, Rader T (2013). Folic acid and folinic acid for reducing side effects in patients receiving methotrexate for rheumatoid arthritis. Cochrane Database Syst Rev.

[21] Ravelli A, Migliavacca D, Viola S, Ruperto N, Pistorio A, Martini A (1999). Efficacy of folinic acid in reducing methotrexate toxicity in juvenile idiopathic arthritis. Clin Exp Rheumatol.

[22] Alsufyani K, Ortiz-Alvarez O, Cabral DA, Tucker LB, Petty RE, Malleson PN (2004). The role of subcutaneous administration of methotrexate in children with juvenile idiopathic arthritis who have failed oral methotrexate. J Rheumatol.

[23] van Dijkhuizen EH, Pouw JN, Scheuern A, Hugle B, Hardt S, Ganser G (2016). Methotrexate intolerance in oral and subcutaneous administration in patients with juvenile idiopathic arthritis: a cross-sectional, observational study. Clin Exp Rheumatol.

[24] Culy CR, Bhana N, Plosker GL (2001). Ondansetron: a review of its use as an antiemetic in children. Paediatr Drugs.

[25] Fedorowicz Z, Jagannath VA, Carter B (2011). Antiemetics for reducing vomiting related to acute gastroenteritis in children and adolescents. Cochrane Database Syst Rev.

[26] Cheng A (2011). Emergency department use of oral ondansetron for acute gastroenteritis-related vomiting in infants and children. Paediatr Child Health.

[27] Kempinska A, Benchimol EI, Mack A, Barkey J, Boland M, Mack DR (2011). Short-course ondansetron for the prevention of methotrexate-induced nausea in children with Crohn disease. J Pediatr Gastroenterol Nutr.

[28] Blanco R, Gonzalez-Gay MA, Garcia-Porrua C, Ibanez D, Garcia-Pais MJ, Sanchez-Andrade A (1998). Ondansetron prevents refractory and severe methotrexate-induced nausea in rheumatoid arthritis. Br J Rheumatol.

[29] Lexicomp Online®, Pediatric & Neonatal Lexi-Drugs®, Hudson, Ohio: Lexi-Comp, Inc.; 2015.

[30] Silverman E, Mouy R, Spiegel L, Jung LK, Saurenmann RK, Lahdenne P (2005). Leflunomide or methotrexate for juvenile rheumatoid arthritis. N Engl J Med.

[31] Benucci M, Saviola G, Manfredi M, Sarzi-Puttini P, Atzeni F (2011). Cost effectiveness analysis of disease-modifying antirheumatic drugs in rheumatoid arthritis. A systematic review literature. Int J Rheumatol.

[32] Beukelman T, Patkar NM, Saag KG, Tolleson-Rinehart S, Cron RQ, DeWitt EM (2011). 2011 American College of Rheumatology recommendations for the treatment of juvenile idiopathic arthritis: initiation and safety monitoring of therapeutic agents for the treatment of arthritis and systemic features. Arthritis Care Res (Hoboken).

